# The beneficial role of companion animals in translational pain research

**DOI:** 10.3389/fpain.2022.1002204

**Published:** 2022-09-05

**Authors:** B. Duncan X. Lascelles, Dottie C. Brown, Michael G. Conzemius, Marie Gill, Michael L. Oshinsky, Michelle Sharkey

**Affiliations:** ^1^ Translational Research in Pain, Department of Clinical Sciences, College of Veterinary Medicine, North Carolina State University, Raleigh, NC, United States; ^2^ Comparative Pain Research and Education Center, College of Veterinary Medicine, North Carolina State University, Raleigh, NC, United States; ^3^ Thurston Arthritis Centre, UNC School of Medicine, Chapel Hill, NC, United States; ^4^ Center for Translational Pain Research, Department of Anesthesiology, Duke University, Durham, NC, United States; ^5^ Global Efficacy & Model Development, Elanco Animal Health, Greenfield, IN, United States; ^6^ Clinical Investigation Center, College of Veterinary Medicine, University of Minnesota, St. Paul, MN, United States; ^7^ National Institute of Neurological Disorders and Stroke/National Institutes of Health, Bethesda, MD, United States; ^8^ Center for Veterinary Medicine Food and Drug Administration, Rockville, MD, United States

**Keywords:** canine, model, pain, translational, drug develoment process

## Abstract

The use of spontaneous painful disease in companion pet animals has been highlighted as one of the changes that could be made to help improve translation of basic science to new therapeutics, acting as a bridge between preclinical and clinical studies, with the goal of accelerating the approval of new therapeutics. This review focuses on the utility of companion pet dogs for translational research by reviewing what outcome measures can be measured, and importantly, the relevance of these outcome measures to human translational research. It also details the practical considerations involved in incorporating companion dogs into human therapeutic development.

## Introduction

This review is the product of the presentations and discussion at an open meeting held at the National Institutes of Health (NIH) in 2017 and 2021 which focused on discussing the current state of expertise in measuring chronic pain in pet animals (1) from the perspectives of both improving clinical veterinary medicine, and contributing to translational pain research. The use of spontaneous painful disease in pet animals has been highlighted as one of the changes that could be made to help improve translation of basic science to new therapeutics, acting as a bridge between preclinical and clinical studies, with the goal of reducing the failure rates of human clinical trials, thus accelerating the approval of new therapeutics (2–4). This current opinion review focuses on the utility of companion pet dogs for translational research by reviewing what outcome measures can be measured, and importantly, the relevance of these outcome measures to human translational research. It also details the practical considerations involved in incorporating companion dogs into human therapeutic development.

## Problem with traditional translational research paradigms for persistent pain and where pet dogs may fit in

**Figure 1:**
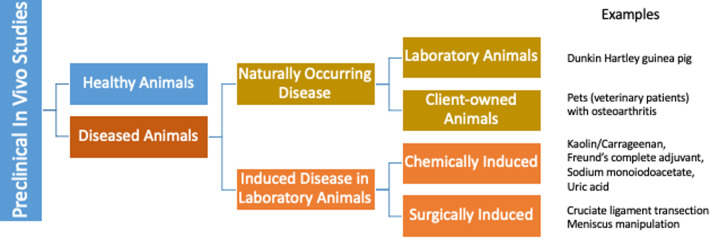
The majority of preclinical models of osteoarthritis are induced (i.e., created) to model or mimic the target clinical conditions. Naturally occurring osteoarthritis does exist, both in laboratory animals, and in owned pet animals.

**Figure 2:**
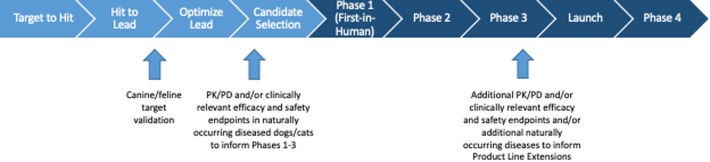
Schematic of the human drug development process, with indications as to where studies in companion animals may contribute to and enrich the translational paradigm.

In the US more than 100 million people suffer from persistent pain with an economic impact of $600 billion annually, at least one third of which is attributed to arthritis and other musculoskeletal pain (5). While existing therapies for chronic, persistent pain have significant limitations, the current practice of translational biomedical research is not producing novel therapeutics to address this unmet need (6, 7). Consequently, the induced rodent models and the outcome measures used in pain research have come under scrutiny with proposals for refinement of current models (8) as well as development of new models and outcome measures that are more directly applicable to prevalent painful conditions (9, 10). The majority of preclinical models are “induced” (i.e., created) to “model” or “mimic” the target clinical conditions (Figure 1). These induced models have worked well for increasing mechanistic understanding of disease, but do not appear to have worked as well for selecting new analgesic drug candidates (11). One approach to improving translation is focusing some efforts on studies in animals with *spontaneous disease conditions* that parallel the human disease, alongside the induced rodent models (Figure 2).

Companion pet dogs offer a unique opportunity to assess chronic pain states in a physiologically relevant “model” of the disease (3). The dominant symptom of musculoskeletal disease for both humans and dogs is pain, and the current therapeutic goal for both species is management of that pain and associated loss of function. To capture data on clinically and translationally relevant pain severity and the impact of pain data in pet dogs, a variety of outcome measures have been developed, validated and tested. Across many classes of compounds in which there have been studies of chronic pain conditions in companion animals and the same conditions in humans, the analogous results have been seen (Table 1) (12–17). In addition, many of the drugs used to treat pain in people are clinically used off-label to treat pain in dogs as well.

**Table 1:** Although a relatively small body of comparative work, it can be seen that across all classes of compounds in which there have been studies in pet animal chronic pain conditions (osteoarthritis, osteosarcoma) and the same condition in humans, the same conclusions have been observed, but not always agreeing with the body of work in rodents.

Drug	Efficacy in rodents	Efficacy in dogs	Efficacy in humans	References
NSAIDs	Yes	Yes	Yes	(15, 18)
Anti-NGF mAbs	Yes	Yes	Yes^a^	(12, 13)
TRPV1 antagonist	Yes	No	No	(16, 19)
Resiniferatoxin	Yes	Yes	Yes	(17, 20, 21)
Substance P-saporin	Yes	Yes	(Awaiting results)	(22, 23)
NSAID EP4 receptor antagonist	Yes	Yes	(Ongoing)	(24, 25)
Capsaicin (intra-articular)		Yes	Yes	(26)

This table is a high overview of the predictive utility.

^a^ The first anti-NGF mAb for humans has recently been declined a marketing authorization by the FDA on the basis of only modest efficacy and side-effects; in contrast, anti-NGF mAb products are now approved for use in dogs and cats in several countries across the globe.

## Rationale for incorporating pet dog studies into translational research

Common human conditions like cancer, and cardiovascular, neurologic and inflammatory diseases similarly occur in dogs, with similar clinical signs (27, 28). The One Health Initiative (29) recognizes that human and animal health and the health of the environment are intimately linked. Using companion pet dogs as a natural model for studying human conditions may better reflect the complex genetic, environmental, temporal and physiological influences present in humans compared to purpose bred laboratory animals studied in a controlled environment.

Companion animals with naturally occurring long-standing (chronic) pain due to cancer, osteoarthritis (OA), and intervertebral disc degeneration (IVDD) have disease similarities to the human disease counterpart. In humans, lower back pain (LBP), including LBP due to IVDD, is a leading cause of disability. IVDD also develops spontaneously in dogs in the caudal lumbar region (“lower back”) with similar clinical signs as humans. Naturally-occurring IVDD is common in chondrodystrophic dogs, which could serve as a disease model for evaluating human IVDD pathophysiology and therapies. Conducting a clinical study in dogs with IVDD, in accordance with similar NIH clinical research standards and comparable study size as a human clinical trial, is feasible (30). Other similarities between human and naturally-occurring canine chronic pain conditions are outlined below, but there are also limitations with companion animal models that should be considered. For example, factors that may influence the translational utility of using a canine model include neutering status, breed variation, 40-fold size difference among dog breeds, quadrupeds versus bipedal humans, and shorter canine lifespan.

## The role of pet animal studies: Go/no-go decisions

In analgesic therapeutic development, pre-clinical testing is used to both establish whether the putative analgesic is associated with efficacy, and establish a toxicity profile (https://www.fda.gov/patients/drug-development-process/step-2-preclinical-research). A drug product's pre-clinical profile is crucial to making Go/No-Go (GNG) decisions. Developing drugs is time consuming and expensive (31, 32), therefore, improved GNG decision making about whether to move forward with a new product or stop development is an important evaluation with significant financial implications. Generally, pre-clinical work is done in healthy laboratory animals in order to predict a drug's toxicity (safety profile) in healthy human volunteers, who will be initially exposed to the drug in Phase I studies. Early proof of concept (POC) studies to assess efficacy are usually performed in rodent laboratory animal models of induced disease. However, these induced models often do not reflect the target clinical disease state, and the standard outcome measures (e.g., reflexive testing) do not reflect the dimensions impacted by pain in human patients (e.g., mobility, pain at rest, affective component of pain) (33). Efficacy evaluation(s) in pet dogs with persistent pain may be more predictive of how a drug will perform in Phase II and III human clinical trials (3), although currently there is little to substantiate this suggestion (see Table 1). Additionally, POC studies may provide insight into potential side effects and novel, appropriate endpoints for assessing this specific type of pain.

Drug efficacy research in non-rodent animals prior to testing in humans is not required in human drug development prior to FDA approval. However, it may provide critical information to optimize the GNG decision. In a pre-clinical assessment guidance for industry pertaining to human cellular and gene therapy products (https://www.fda.gov/media/87564/download), the FDA notes that assessing the activity and safety of some products in animal models of disease or injury may be preferable to using healthy, purpose bred animals.

## Clinically relevant outcome measures are available

Pain impacts many dimensions in people (physiologic, sensory, affective, cognitive, behavioral, and sociocultural) (34). However, pain does not necessarily impact each of these dimensions to an equal degree, and indeed the same painful condition may impact these dimensions differently from individual to individual. The factors that shape how pain impacts these different dimensions are unknown, but the environmental and temporal similarities between spontaneous disease in pet dogs and humans may make the spontaneous pet animal models more relevant than rodents. To take advantage of this complex model, outcome measures for the various dimensions impacted by pain are needed. For several naturally occurring painful diseases (primarily musculoskeletal pain associated with osteoarthritis) in pet animals, the outcome measures are well developed, and the outcomes are relevant to humans, these are discussed below.

## Relevant outcomes can be measured in companion pet dogs with osteoarthritis pain

### Clinical metrology instruments

A Clinical Metrology Instrument (CMI) is a sequence of questions relating to a clinical disease or process, and responses are based on subject experiences or proxy assessments. CMIs are available for patients, clinicians, parents and pet owners to complete. When the focus is on patients, they are often referred to as patient reported outcome (PRO) measures and these are commonly used as clinical trial endpoints in human drug development. The PRO measure comes from the patient as a measure of how the patient feels or functions *via* self-reporting or by interview. There is both academic (35) and regulatory (https://www.fda.gov/drugs/drug-development-tool-ddt-qualification-programs/clinical-outcome-assessment-coa-qualification-program) agreement that such metrology instruments should be developed using sound psychometric principles, and tested rigorously for validity. The FDA/COA qualification program provides consultation on endpoint development, validation, interpretation, and representation in labeling and advertising. There are at least 78 PRO measures with varying levels of validity described in recent literature to assess OA in humans; and PRO measures have been used in assessing human OA for over 35 years (36).

Animals cannot self-report; however, the pet owner can make a proxy assessment of the impact of chronic pain, just as a parent makes a proxy assessment of their child. Such owner assessments have been developed using the same methodology used for human CMIs. They have been validated, and successfully used in numerous randomized, placebo-controlled, blinded, clinical veterinary studies aimed at assessing the utility of chronic pain therapeutics. The individual instruments are different and capture different information across varying domains impacted by pain (1) similar to the recommendations for measuring human chronic pain (35). These owner-completed CMIs can be used in studies utilizing pets with naturally occurring chronically painful disease and serve as a preclinical evaluation to determine whether the biological effect of a proposed human analgesic can be demonstrated *in vivo*.

Several instruments based on owner assessment of chronic pain and pain related function in dogs (1) have been validated. There still are outstanding questions around the appropriate use of these instruments and interpretation of the results; these challenges are similar to the challenges of using CMIs in human clinical trials (37). Some of the challenges include:

• Most current CMIs have been developed to assess OA-pain and validated using systemically administered NSAIDs. Development of CMIs across other pain conditions and testing using a variety of analgesic classes is needed.
• There should be uniformity across studies in how these instruments are used to allow for comparative interpretation of results, and this likely requires the publication of detailed guidelines on the administration and interpretation of CMI data.
• There are translation and linguistic validation issues to consider especially when conducting a global clinical trial in different countries.
• There are challenges designing efficacy studies using proxy CMIs in a way that minimizes potential biases and placebo effects inherent in the use of subjective outcome measures.
• The validity of electronic versions of CMIs that were originally paper. Changing to an electronic format or use in a different setting may alter the responses obtained (38), however, these differences may be small (39) and electronic questionnaires administered in certain contextual environments may result in better data than paper forms (40).
• Work needs to be performed to understand what changes on CMI scores represent a clinically meaningful change in the animal, similar to the discussions around human CMI data (41–43).

### Physical activity monitors

Physical Activity monitors (PAMs) based on accelerometry (± gyroscope and magnetometer) provide an opportunity to investigate patient activity in their natural environment in a non-invasive manner over an extended period of time. Several studies evaluating the use of accelerometers in dogs have associated activity counts from these accelerometers with the actual behavior and movements of the animals (44–50), and recent work has shown how activity monitors can be used to estimate distance travelled (51). With the simplistic premise that chronic joint pain will result in decreased activity, and alleviation of that pain will restore activity, several studies have used activity monitors as an objective outcome measure to assess putative analgesics (50, 52, 53). However, this simplistic approach may not be sensitive to the changes in activity induced by pain relief. For example, with pain relief, rest times may be more restful, while the intensity of activity may increase. Such simultaneous bidirectional changes in activity may not be detected simply summing or averaging activity. The simplistic approach using totals or averages of activity is now being refined, and high-frequency longitudinal activity data collected by accelerometers are being analyzed in ways that allow for detection of changes in patterns of activity (54, 55). In addition to advancing statistical approaches, ongoing work is evaluating factors, such as the relationship between the center of mass and the PAM, that may contribute to the large variance in data (56).

Activity monitors provide objective data on movement in both animals and humans, however it must be remembered that to a large extent, owners of dogs define the amount of activity that their dogs undertake, and so “objectively” measured activity can be biased by owner expectations of treatment effects. Owners can promote or limit activity based on their assumption that their dog received active or placebo—leading to placebo effects that we would not expect from a truly objective outcome measure. Direct translation of AM technology between bipeds and quadrupeds should be avoided. Bipeds have a relatively predictable step pattern or gait (left-right-left) and while their gait changes with speed (51, 57), the order of foot falls remains predictable. In contrast, quadrupeds’ gait and order of foot falls can change (walk, pace, canter) with different speeds. Thus, mathematical PAM algorithms designed for people cannot be directly applied to quadrupeds, and vice versa. Second, patient morphometrics need to be considered when evaluating PAM data both within, and between, species. For example, the limb length of a Great Dane may be five times that of a Pomeranian; in contrast, limb length within humans is more uniform. Also, the specific outcome generated by the PAM may be of differing importance between different species. In a recent study of people with knee OA, it was reported that step counts, and energy expenditure had the strongest associations with functional assessment (58). This may be because the vast majority of human motion is lying, sitting, standing and various speeds of forward motion. While these are also common motions in veterinary patients, it is not unusual to see a dog perform high energy activities (e.g., jump from a height that equals their own) that would generate a g-force 2–3 times that of walking; these types of activities would be rare for a human to perform. The frequency of these activities may be impacted by disease (or treatment); so, it may be more important to document g-force in veterinary patients rather than simply reporting step count.

The premise that human and veterinary patients are negatively impacted by chronic pain and are less active than their healthy counterparts has been accepted (1, 58). However, in a study of people with OA, it was reported that, “the impact of chronic pain on everyday physical activity was relatively small” (59). This was a similar conclusion to another study that compared objectively measured movement-related activity in healthy controls and OA patients, and found that controls had significantly higher movement-related activity than OA patients; albeit, only by 2.9% (60). However, it is important to emphasize that relatively little work has been performed employing PAMs in human chronic pain studies, and thus far data analysis has been very simplistic. A lack of self-report by animals has probably created more emphasis on developing surrogate objective outcome measures, but if these are to be used in translational pain studies, the relevance to humans needs to be better understood. This means more work being done in humans with accelerometry in relation to pain may be necessary. A recent publication describing research priorities for chronic pain in animals (61) identified several limitations of PAMs. Many of these limitations exist regardless of the species and provide research opportunities so that PAM use and reporting can be better translated between and across all patients that suffer from chronic pain.

### Gait analysis

**Figure 3:**
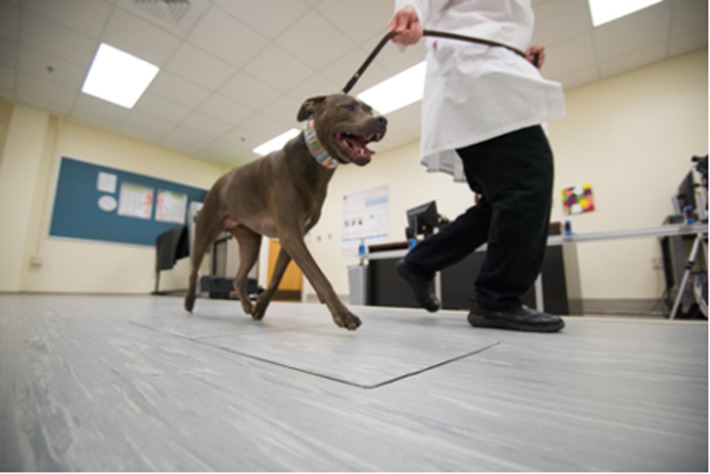
Limb use can be very accurately measured in dogs, just as in humans, using force platforms. The photograph shows a pet dog being gaited over dual in-series AMTI force platforms mounted in the center of a walkway to measure limb use and how this is affected by joint pain. Image copyright belongs to NC State University.

Utilizing gait analysis as an outcome measure has been common in animals and people for decades. The greatest translational opportunity is with measuring ground reaction forces (GRFs) *via* force or pressure platform gait analysis in dogs. There are subtle differences in the technical aspects of measuring GRF in dogs and people. First, as quadrupeds, dogs can step on a force plate with more than one foot while walking (62). To limit this, dogs weighing greater than ∼20 kg are studied when using a standard (e.g., AMTI) force platform because they have a stride length that is adequate for a single foot strike at a time on the force plate (Figure 3). Alternatively, a pressure platform or walkway can be used for dogs of all sizes since GRFs are captured for each foot over time. Second, dogs are generally unaware of the location of the force platform in a walkway. This is advantageous as their gait remains unchanged during each trial, but as such, additional trials may be needed to collect appropriate data. Finally, software specific for quadrupeds is used. Despite these differences, the fundamentals of kinetic gait analysis are identical, and the equipment used in human and veterinary studies is identical (e.g., AMTI force platform or Tekscan walkway pressure platform). In both species GRFs [peak vertical force (PVF), vertical impulse (VI), rising and falling slopes] are evaluated and there is a need to control velocity and acceleration, define a valid trial, and establish how the data will be evaluated (e.g., multivariate analysis, evaluation of asymmetry, average of multiple trials, improvement in group means versus individual subject compared to own baseline). Best practices for measuring and reporting GRFs in dogs have recently been published (63).

The parallels of kinetic gait analysis in dogs and people are not limited to the technical aspects of the outcome measure. While changes in kinetic gait can be from a mechanical change in gait (e.g., one limb significantly shorter than the other; functional limb abnormalities), once those are eliminated from a study population with joint pain, it is reasonable to suggest that gait analysis serves as a good, and objective, indirect estimate of movement-associated pain. While GRFs are not intrinsically a measure of pain, they can, in the correct phenotype, be used as a proxy measure of pain. Based on this premise, GRFs have been used for the evaluation of pain secondary to spontaneous osteoarthritis (OA) of the stifle (knee), hip and elbow in client-owned dogs, and the mitigating effects of treatments (64–68). The modulating effects of analgesic treatments on GRFs in dogs with joint pain support the assertion that in this scenario changes in GFRs are reflective of a change in movement-associated pain. Similar work using measured GRFs has been performed in humans with knee OA (69–71). Measured GRFs in relation to pain likely have direct translational utility between veterinary patients and humans.

### Quantitative sensory testing

Quantitative sensory testing (QST) is a psycho-physical test used to quantify somatosensory function (72). Evaluation of the response to externally applied physical stimuli, such as pressure, heat, or cold is used to provide information regarding the functioning of the sensory system. QST employs the application of one or more modalities (e.g., mechanical, thermal) of stimuli. The stimuli are of graded intensity (e.g., measurable force or temperature) applied as a fixed stimulus, or a ramped stimulus, and may represent either noxious or non-painful levels of stimulation. The end-point of a particular test is the evoked somatosensory response (Suokas et al., 2012); in humans this will most commonly be a verbal report, whilst in animals behavioral indicators are assessed (72).

Recent work has shown that QST assessed changes are similar in both humans and dogs with OA. Human patients with OA, when compared to healthy controls, demonstrate widespread hyperalgesia, facilitated nociceptive temporal summation, and decreased activity of the descending endogenous analgesic system (73–76). Similar somatosensory changes have been found in dogs (77–79) affected by naturally occurring OA. Additionally, reversal of QST-assessed hyperalgesia after total joint replacement was found in dogs with hip OA undergoing total hip replacement (80), as found in humans after total hip (81) or knee (82) replacement. Relatively little work has been performed in humans to evaluate analgesic efficacy using QST, although recently NSAIDs were found to have minimal effects on the descending endogenous analgesic system (83) and similar findings were recently reported in dogs with OA administered an NSAID. Some work in humans has focused on using QST to define phenotypes and predict analgesic efficacy in these phenotypic populations (84, 85) and it is likely that the same approach could be used in pet animals. There is a lot of potential for translational utility of QST as a modality, but there are differences in the measures in humans and animals. Humans can describe the sensation associated with various stimuli, and measures of “first detection”, “first pain”, and “maximum tolerated pain” can be measured, whereas the endpoint in animal studies is based on a reaction or withdrawal. As in human studies, there is a need for standardized methodology across studies.

### Nociceptive withdrawal reflex

The NWR is typically measured by recording the electromyographic (EMG) response to electrical stimulation of a peripheral nerve (86). In humans, there is a robust close correlation between the threshold current intensity required to elicit the NWR and the subject's pain threshold leading to the interest in the use of the NWR in pain research. Facilitation of the NWR along with an increase in subjective pain sensation have been shown in human models of secondary hyperalgesia (87) including OA (88–90) suggesting that the augmentation of the NWR is a useful and objective biomarker of central sensitization. Methodology has been developed in research dogs (91–93) and its use in pet dogs with naturally occurring OA has been described, although the need to heavily sedate canine patients may limit the utility of NWR as an outcome measure (94). As yet, NWR has not been used in either humans or animals to test the antihyperalgesic or analgesic effects of analgesics, and the clinical relevance of NWR results is relatively undefined.

### Other outcome measures

There have recently been calls for development and employment of an expanded set of outcome measures in human OA, including objective measures (95). In veterinary medicine, the literature often includes, and indeed often relies upon, objective measurements due to the lack of self-report. The outcome measures recommended in humans can all be measured in dogs with OA pain, underlining the translational utility of these spontaneous models. Additionally, other dimensions impacted by chronic pain have the potential to be measured in the context of canine OA pain. In humans, chronic joint and musculoskeletal pain has a negative impact on cognitive function (96, 97). Although not discussed at PAW 2017, measures of cognitive function in dogs have been developed, and used in pet dogs (98).

## Cancer pain outcome measures in dogs

Biological similarities between canine and human osteosarcoma (OSA) makes the dog a promising model for translational cancer pain research. Similarities include matching biological behavior, similar response to treatments, almost identical histological characteristics, and shared global gene expression signatures (99, 100), yet there is a >10-fold increased incidence of canine OSA compared to human OSA. Just as in humans, canine OSA is associated with pain (101, 102) and research shows canine OSA cells express nociceptive ligands (103). Most OSA is appendicular and therefore, all the outcome measures that have been developed for canine OA can likely be applied to canine OSA (104). Indeed, limb use following radiation therapy for limb OSA has been assessed using objective force plate measurements (105). The extended course of disease, compared with rodent models, allows for clinically relevant efficacy data collection from appropriate numbers of subjects, and the shorter overall lifespan of dogs, compared with humans, provides a time course of disease within a time frame reasonable for data collection. The canine OSA model has been integral in moving certain compounds forward to human clinical trials, for example, substance *P*-saporin (ClinicalTrials.gov identifier: NCT02036281) (17, 106).

## Neuropathic pain outcome measures

It has become clear that neuropathic pain can be present in many conditions, such as cancer and chronic inflammatory conditions, all of which can cause varying degrees of physical damage to the nervous system. Neuropathic pain affects up to 90% of people with chronic spinal cord injury (SCI), and SCI is also common in veterinary patients (107). The QST methodology developed in dogs with OA has been applied to breeds where SCI following intervertebral disk extrusion commonly occurs (108). QST methodology may allow for the measurement of altered somatosensory processing associated with SCI to act as a surrogate measure of neuropathic pain. However, these measures have the added complexity that apparent altered sensation following SCI may be due to an inability of the system to function due to physical interruption, compared to a dysfunction of an anatomically intact system (such as is the situation in osteoarthritis), and this has to be carefully navigated in animals who cannot describe sensations. Indeed, one of the hurdles to developing outcome measures for neuropathic pain in pet dogs is the heavy reliance on verbal descriptors in humans to describe and diagnose neuropathic pain. Despite this, several conditions in dogs do appear to mimic neuropathic pain conditions in humans. Chiari-like malformation in Cavalier King Charles Spaniels has long been considered to be associated with neuropathic pain and headache (109), and recently attempts have been made to develop owner-based questionnaires (110, 111) and QST (112) to measure this pain. Intervertebral disk disease pain, nerve root entrapment, nerve root tumors and lick granulomas are all considered to be neuropathic pain conditions, but development of outcome measures has not been comprehensively explored yet.

## Practical considerations when using pet animal studies in “human” therapeutic drug development

Investigation of naturally occurring pain conditions in companion pet dogs may provide clinically relevant endpoints, using outcome measures as discussed above, that closely resemble endpoints in human randomized clinical trials (RCTs), and, importantly, outcome measures that are relevant to humans. In many circumstances this translational opportunity provides a closer perspective to human pain conditions compared to assessing pain induced behaviors in laboratory species (113, 114). Therefore, this strategy can be considered bridging, in contrast to a direct jump from induced animal pain studies to clinical trials in humans. There are factors to consider when conducting a study in veterinary patients that suffer from persistent pain (e.g., OA) to ensure study results are valid, and the studies are conducted appropriately.

**Figure 4:**
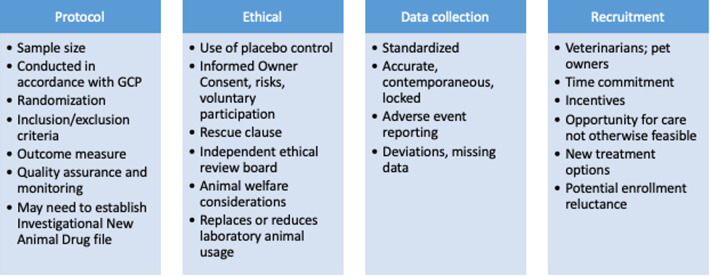
Clinical study design considerations for companion dog studies of persistent pain as proof of concept for human analgesic development.

Randomized clinical animal studies can be designed and conducted to comparable rigor of human RCTs. The design of studies involving pet animals can, and should, include randomization, blinding, appropriate controls and quality assurance (Figure 4) (115). In addition, the protocol should include the description of outcome measures, a specified *a priori* definition of success, assessment of adverse events, and masking procedures (116). Optimizing study power is a common challenge that can be met by designing a multi-institutional study. Working with a statistician, running a study in accordance with Good Clinical Practices (GCP) and consulting with a Contract Research Organization (CRO) prior to the onset of the study should be considered. All of these study design factors are possible to accomplish, and there are several CROs that specialize in conducting veterinary studies that can help facilitate these aspects. Unlike human medicine (clinicaltrials.gov), there is no requirement to register clinical trials in veterinary species. There is an optional registry run by the American Veterinary Medical Association (AVMA) (https://ebusiness.avma.org/aahsd/study_search.aspx).

Many pet owners expect and seek high level veterinary care and are willing to have their pet participate in a clinical study (117). Informed consent from pet owners is critical and should include an accurate description of all potential patient risks, benefits, potential benefits to human health, the likelihood of their pet being randomized to an investigational treatment group versus a placebo group, the voluntary nature of study participation, the ability for owners to withdrawal their pet from a study without prejudice, and description of a rescue or escape clause. In order to communicate the risks, safety data generated in dogs at the dosing regimen being proposed for the proof of concept study needs to be available. This has often already been generated as part of the work performed for the Investigational New Drug (IND) application. It goes without saying that dogs are larger than rodents, and significantly more active pharmaceutical ingredient (API) is needed for dog studies. Study sponsors are expected to cover all the costs associated with the study, and other incentives for participation can be built in, but should not be used to pressure owners into having their pets participate in studies that inherently have some risks. The requirements for oversight of such studies vary from country to country. In the UK, for example, randomized, placebo-controlled clinical studies require Home Office approval. These requirements are very appropriate, and protect the owners, pets, investigators and sponsors involved in the study. Although regulations may differ in other countries, the authors are adamant that the client consent form, the study design, and background information should be reviewed by an institutional ethical review board or some independent party that does not have a conflict of interest to ensure the proposed study is ethical and justified. Part of ethical review should be to ensure the study is designed and powered appropriately to avoid statistical errors.

A veterinarian should always be involved with and overseeing the care of the individual animals participating in any study. There are a variety of well-equipped veterinary sites with the expertise to participate in well-controlled pet animal studies. Many veterinary facilities have the same diagnostic and procedural capabilities as human hospitals. The Clinical and Translational Science Award One Health Alliance (COHA: https://www.ctsaonehealthalliance.org/) is a network of veterinary medical schools (some associated with CTSA medical schools) that research naturally occurring diseases that affect animals and humans (e.g., cardiovascular disease, cancer, arthritis, etc.) (3). One intent of COHA is to establish a network of facilities for conducting pet animal clinical studies according to a set of standards pertaining to animal welfare, data collection, quality control and GCP. In addition to private and public veterinary medical schools, numerous private veterinary hospitals across the country have the expertise to conduct high quality research.

Cost and speed of completion of such studies are common questions that come up early in the discussion of considering these translational proof of concept studies. The number of animals required, the duration and the complexity of the study drive the cost. As an example, a typical OA pain study, involving screening and evaluation of a therapeutic over several months, will likely cost $2,000 to $6,000 per subject, inclusive of all personnel and study related costs. Sample size estimations should be based on the specific therapeutic, therapeutic class, route of administration, enrolled phenotype, and outcome measures employed. However, as an example, a study evaluating an intra-articular therapeutic, with an expectation of ∼5.0% body weight change in PVF (and SD of 4.5), would require ∼19 subjects per group for a study power of 0.9. Success of a study such as this relies on careful selection of the appropriate phenotype. A study evaluating a systemic analgesic equivalent to an NSAID, with an owner-evaluated outcome, would require larger sample sizes. For example, using the largest publicly available data set (18), and the CBPI as an outcome measure, and assuming success response rates of 0.51 with an NSAID, and 0.25 with placebo, 50 dogs in each group would have a study power of 80%, and 65 dogs in each group would have a study power of 90%. These are provided just as examples, but the authors emphasize the need to calculate sample sizes based on the particular circumstances of the therapeutic, study and aims.

Electronic data capture can decrease the time to first data read out, and is commonly used in studies of pet dogs. As the number of animals required increases, having the study conducted at multiple sites reduces enrollment time. In general, the experience of the authors is that drop-out rates are low, with owners who agree to having their pet in a study being engaged because of the potential benefit to their pet, or to other pets and people (117).

Such studies are already being performed by human pharmaceutical companies (118), and also by NIH-funded research teams (17, 20, 106).

## The role of companion pet animals for discovery/reverse translation

Drug development programs will fail if their biological basis is not sound. Whether or not basic science work is relevant to a clinical condition will depend on whether or not the clinical condition shares the same neurobiological mechanism being investigated in the model. In this respect, evaluation of tissue from naturally occurring disease states may provide information about the *neurobiology of pain in the natural disease state*, and thus help to define potential targets. There is growing interest in this general approach—so called *reverse translation*, or maybe better, *multidirectional translation*, with neurobiological evidence from the target condition being used to inform basic mechanistic research (119). Recent studies of human dorsal root ganglia (DRG) tissues clearly show significant neurobiological differences from rodents (120), suggesting that assumptions about target validity based on rodent neurobiology should be performed carefully. Tissue is not always easily obtained from humans, however peripheral tissue can be readily obtained from the millions of joint surgeries performed each year on pet dogs (121), and central nervous system tissues may be available *via* owner consent from the thousands of dogs with OA-associated pain that are euthanized each year. If the outcome measures described above are used to phenotype the pain in pets, then the information garnered from such tissue can point to novel relevant pathways, as has been recently demonstrated (122, 123). Because of the recognized utility of naturally occurring disease in large animal models (e.g., horses, cattle, sheep, goats, pigs and dogs), there has been a recent call to develop *in vitro* and *ex vivo* techniques similar to those used in rodent work, to extend the utility of large animal models (124).

## Benefits to canine health

While the thrust of this review has been to discuss the potential utility of studying pain in companion animals, dogs in particular, to inform and facilitate analgesic therapeutic development in humans, there are significant potential benefits for canine health. Information gathered in the course of “proof of concept” studies, performed in companion dogs with naturally-occurring disease, invariably creates information on how to control pain in companion pet dogs. Such information, combined with the safety data that is often generated from work in dogs, can form the rationale for a veterinary therapeutic development program. The approval process for veterinary products is generally overseen by the FDA Center for Veterinary Medicine.

## Conclusion

In the last 25 years, much progress has been made in developing methods to measure chronic pain *via* subjective and objective methods in pet animals. Most work has been focused on chronic joint pain conditions with some work in other areas of chronic pain such as neuropathic pain and cancer pain. These advances in measurement have facilitated consideration of the use of these naturally occurring chronic pain models in translational research, primarily to inform researchers of the potential utility of therapies for human chronic pain treatment. Naturally occurring chronic pain conditions in pets may better reflect the complex genetic, environmental, temporal and physiological influences present in humans compared to purpose bred laboratory animals in a controlled environment. Harnessing information from studying responses to putative therapeutics in these models may help to make translational pain research more efficient and successful.
